# Sports Nutrition: Current and Novel Insights (2nd Edition)

**DOI:** 10.3390/nu18121896

**Published:** 2026-06-11

**Authors:** David C. Nieman

**Affiliations:** Human Performance Laboratory, Department of Biology, North Carolina Research Campus, Appalachian State University, Kannapolis, NC 28081, USA; niemandc@appstate.edu; Tel.: +1-828-773-0056

The field of sports nutrition is advancing rapidly, propelled by innovations in molecular and human systems biology, multi-omics technologies, and applied performance science. Present-day research increasingly emphasizes precision nutrition, inter-individual variance in response to dietary interventions, and the integration of nutrition with body-wide physiological systems and processes including the gut microbiome, immunity, metabolic/energy functions, endocrine and nervous integration and regulation, and musculoskeletal function and recovery. These themes were highlighted in the first edition of *Sports Nutrition: Current and Novel Insights* and have been further expanded in this second edition, alongside emerging perspectives discussed in recent publications [1,2,3,4,5,6,7].

Collectively, the six articles published in this Special Issue of *Nutrients* provide a multifaceted view of the current state of sports nutrition science. These span ergogenic supplementation with a focus on plant-derived bioactives, dietary knowledge gaps, recovery physiology, and broader health considerations in athletes.

Perkins et al. (2024) (contribution 1) examined the effects of repeated dosing with anthocyanin-rich New Zealand blackcurrant extract on high-intensity intermittent running performance. Their findings reinforce the ergogenic potential of polyphenol-rich supplements, demonstrating improvements in total and high-intensity running distance (~8%). A subset of participants identified as responders exhibited substantially greater benefits. This study underscores substantial inter- and intra-individual variance in responses, an outcome that represents a persistent and enduring challenge in sports nutrition research. Mao et al. (2025) (contribution 2) reported that Brussels chicory, rich in phenolic acids, improved time to exhaustion and enhanced post-exercise recovery, potentially via increased lactate oxidation. Together, these studies highlight the growing interest in plant-derived bioactives as modulators of performance and recovery.

Montalvo-Alonso et al. (2024) (contribution 3) investigated the combined effects of caffeine and sodium bicarbonate on muscular endurance. While caffeine alone improved repetitions, velocity, and power output, the anticipated additive or synergistic benefit of co-ingestion was not observed. These findings emphasize that combining established ergogenic aids does not guarantee enhanced efficacy, stressing the need for strong research designs, a focus on underling mechanisms, and a clear supplement dosing rationale and strategy.

Jagim et al. (2025) (contribution 4) provided nutrition-based insights into adolescent athlete populations, demonstrating that they exhibited poor sports nutrition knowledge and misperceptions of energy and macronutrient requirements. Energy and carbohydrate needs were underestimated and protein intake overestimated by these young athletes. These findings reinforce the importance of nutrition-based guidance by qualified professionals for adolescent athletes. The goal is long-term adherence to evidence-based dietary practices to complement intensive training regimens in young athletes.

Mielle et al. (2025) (contribution 5) explored the rarely investigated intersection of sports nutrition and oral health. This paper revealed high prevalence rates of dental caries and erosion among athletes. These were linked in part to dietary habits such as frequent consumption of sugar- and acid-containing sports products. This work broadens the scope of sports nutrition by highlighting unintended health consequences associated with common fueling strategies among athletes and fitness enthusiasts. Thus, there is a need to consider all aspects of health while providing specific nutrition-based guidance for athletic performance.

Nieman et al. (2025) (contribution 6) examined the effects of hemp fiber supplementation on post-exercise gut permeability and metabolomic responses. Hemp hull fiber derived from the outer seed coat of hemp is rich in bioactives and insoluble fibers. While no changes were observed in gut permeability, significant alterations in metabolic pathways and gut-derived metabolites were detected. This study illustrates the increasing use of omics technologies to uncover subtle but potentially meaningful adaptations to nutritional interventions.

These contributions emphasize several unifying themes. The variability in response to blackcurrant extract and other diet-based interventions reinforces the current emphasis on personalized sports nutrition. When possible, studies should consider and incorporate measurements of responder variance, repeated-measures and crossover designs, and integration with genetic, metabolic, and microbiome data. The inclusion of polyphenols, fibers, and plant bioactives reflects a paradigm shift toward multifunctional nutritional strategies that influence not just performance and recovery but also long-term health. The integration of metabolomics and gut microbiome composition outcomes aligns with broader trends in systems biology. This enables a deeper understanding of how nutrition interacts with immune function, metabolism, and exercise-induced stress. Despite scientific advances, significant gaps remain in translating knowledge to practice, particularly among youth athletes. Educational interventions and access to qualified nutrition professionals remain critical priorities. The link between dietary practices and oral health highlights that performance-focused nutrition strategies should consider broader health implications, reinforcing a holistic approach to athlete care.

## Conclusions

The second edition of Sports Nutrition: Current and Novel Insights advances the field by highlighting both innovation and ongoing challenges. The future of sports nutrition [1,2,3,4,5,6,7] will likely be influenced and shaped by (1) precision and individualized nutrition frameworks integrating omics data and wearable technologies, (2) an expanded focus on the gut–muscle and gut–immune axes, (3) the development of multifunctional foods and bioactive compounds targeting both performance and long-term health, and (4) improved translation strategies, particularly in youth and non-elite populations.

The included studies demonstrate that while ergogenic supplementation continues to evolve, the future of sports nutrition lies in personalization, systems-level integration, and bridging the gap between scientific discovery and practical application.
